# Function, regulation and pathological roles of the Gab/DOS docking proteins

**DOI:** 10.1186/1478-811X-7-22

**Published:** 2009-09-08

**Authors:** Franziska U Wöhrle, Roger J Daly, Tilman Brummer

**Affiliations:** 1 Spemann Graduate School of Biology and Medicine, Albert-Ludwigs-University of Freiburg, Germany; 2 Centre for Biological Systems Analysis (ZBSA), Albert-Ludwigs-University of Freiburg, Germany; 3 Institute for Biology III, Albert-Ludwigs-University of Freiburg, Germany; 4 Cancer Research Program, The Garvan Institute of Medical Research, Australia; 5 St Vincent's Clinical School, University of New South Wales, Australia; 6 Centre for Biological Signalling studies (bioss), Albert-Ludwigs-University of Freiburg, Germany

## Abstract

Since their discovery a little more than a decade ago, the docking proteins of the Gab/DOS family have emerged as important signalling elements in metazoans. Gab/DOS proteins integrate and amplify signals from a wide variety of sources including growth factor, cytokine and antigen receptors as well as cell adhesion molecules. They also contribute to signal diversification by channelling the information from activated receptors into signalling pathways with distinct biological functions. Recent approaches in protein biochemistry and systems biology have revealed that Gab proteins are subject to complex regulation by feed-forward and feedback phosphorylation events as well as protein-protein interactions. Thus, Gab/DOS docking proteins are at the centre of entire signalling subsystems and fulfil an important if not essential role in many physiological processes. Furthermore, aberrant signalling by Gab proteins has been increasingly linked to human diseases from various forms of neoplasia to Alzheimer's disease.

In this review, we provide a detailed overview of the structure, effector functions, regulation and evolution of the Gab/DOS family. We also summarize recent findings implicating Gab proteins, in particular the Gab2 isoform, in leukaemia, solid tumours and other human diseases.

## Discovery of Gab docking proteins - Ten years on

With the increasing isolation and cloning of protein tyrosine kinase (PTK) substrates and association partners in the mid 1990s, a large number of proteins with no intrinsic enzymatic activity were described and termed as adaptor, scaffold or docking proteins [1]. Although these terms are often used interchangeably, adaptor proteins are usually smaller in size and often function as an inter- or intra-molecular bridge between two proteins or within a single protein, respectively, and thereby play an important role in the assembly of larger protein complexes or the stabilisation of certain conformational states. Examples for such adaptor proteins are growth factor receptor bound protein 2 (Grb2) or the 14-3-3 proteins [2,3]. Scaffold and docking proteins, however, contain multiple structural domains and various protein interaction motifs or docking sites and are consequently significantly larger. Furthermore, docking proteins usually contain one or more moieties that mediate their recruitment to biological membranes by protein-protein or -lipid interactions. Due to their size and molecular characteristics, docking and scaffold proteins may act as platforms for the assembly of signalling subsystems as it is exemplified by the pivotal role of the *kinase suppressor of ras *(KSR) scaffold protein in the orchestration of Ras/ERK signalling [4,5]. Indeed, the genes for several scaffold or docking proteins, including KSR, *Daughter of Sevenless *(DOS) and *Suppressor of Clear *(SOC) 1, were identified by genetic screens in *Drosophila *and *Caenorhabditis *as important modifiers of receptor tyrosine kinase (RTK) signalling pathways, long before biochemical and structural studies revealed their true mechanism of action [4-7]. The discovery of the mammalian DOS/SOC-1 orthologues, Grb2 associated binder 1 (Gab1), Gab2 and Gab3, placed Gab proteins among the first docking proteins identified in mammalian signal transduction [8-10]. Since then, it has become evident that Gab proteins are crucial signalling elements employed by a plethora of receptors and the field has gathered significant insights into their structure, function, evolution, regulation and contribution to various human diseases. In this article, we will review these topics with a particular emphasis on the two latter aspects, for which considerable progress has been made since the last comprehensive reviews were published on these docking proteins more than five years ago [11,12].

## Diversity and structure of Gab docking proteins

**Figure 1:**
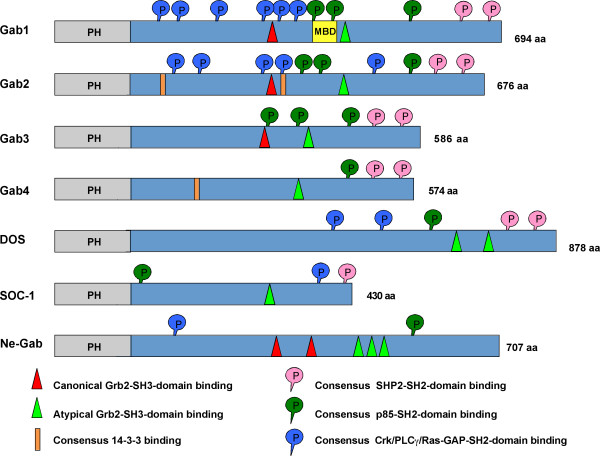
**Conserved structural features of Gab proteins**. Simplified cartoon indicating the modular structure of the human Gab proteins (Gab1-3), the putative human Gab4 protein, DOS, SOC-1 and a putative Gab protein from *Nematostella vectensis *(Ne-Gab). All proteins contain a highly-conserved N-terminal PH domain involved in membrane recruitment. The central proline-rich regions mediate the interaction with SH3 domain-containing adaptor proteins such as Grb2. Consensus motifs for SH2- or PTB domain-containing proteins like SHP2, p85, Crk and PLCγ are indicated. The functionally-characterized 14-3-3 binding motifs in Gab2 and the c-Met binding domain (MBD) in Gab1 are also shown.

The Gab proteins are large scaffold or docking proteins of 50 to 100 kDa found in metazoans [11,12]. Functionally and/or structurally related proteins are: the docking protein FRS2, an important signal transducer downstream of FGF receptors; the IRS proteins that have emerged as critical signalling components regulating insulin action and sensitivity; and the proteins SLP-65 and SLP-76 that fulfil pivotal roles downstream of cell adhesion molecules and antigen receptors and in the haematopoietic system [13-18]. Vertebrates possess at least three paralogues, Gab1 to 3 [8,9,19,20]. In contrast to vertebrates, the genomes of the model organisms *Drosophila *and *Caenorhabditis *contain only one Gab gene [6,7,20-22]. However, comparative analyses of the amino acid (aa) sequences of these invertebrate Gab proteins with the vertebrate proteins, in particular for sequences outside of the highly conserved pleckstrin homology (PH) domain, suggest that SOC-1 probably represents an early divergent member of the Gab family. This issue will be further discussed below. As explained in detail in Fig. 1, all Gab proteins share a similar modular structure, including a PH domain at their N-terminus, proline-rich regions in the central part and multiple phosphorylated tyrosine residues.

## Recruitment of Gab proteins to their site of action

**Figure 2:**
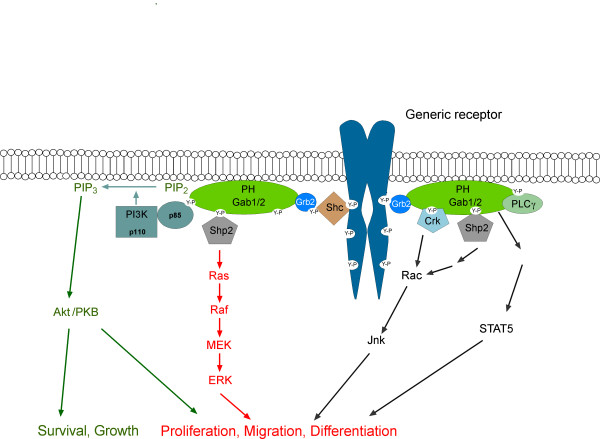
**Recruitment of Gab proteins to activated receptors and the main effector arms of Gab signalling**. The indirect mode of recruitment applies to all receptors except c-Met (see text and Fig. 3 for details). Characteristics of this mechanism are that phosphotyrosine residues within the cytoplasmic tails of activated surface receptors serve as docking sites for the SH2 domain of Grb2, which in turn binds via its C-terminal SH3 domain to specific binding sites in Gab1-3. Alternatively, Shc can serve as additional bridging molecules between Gab and activated receptors. Membrane/receptor association leads to tyrosine phosphorylation of Gab proteins and subsequent recruitment of SH2 domain-containing effectors such as SHP2, p85, PLCγ and Crk. While it has been shown by numerous studies that the association between Gab proteins and the effectors Shp2, p85, Grb2, Crk and PLCγ represents a direct protein-protein interaction, the coupling between Gab and STAT5 needs to be resolved in the future.

**Figure 3:**
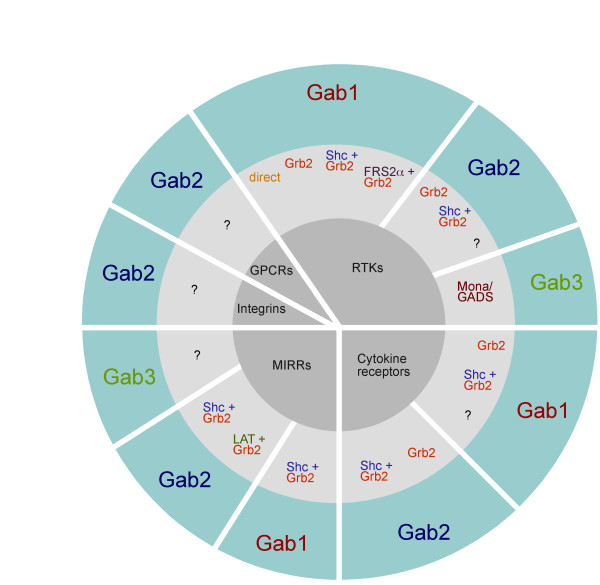
**The Gab recruitment code**. Simplified overview of the various mechanisms utilized for recruitment of Gab proteins by receptor tyrosine kinases (RTKs), cytokine receptors, multichain immune recognition receptors (MIRRs), integrins and G-protein coupled receptors (GPCRs). Inner circle: Receptor classes recruiting Gab proteins. Middle circle: Adaptor proteins involved in recruitment. Outer circle: Gab proteins recruited. For details see main text and references therein.

Docking proteins of the Gab family use several different mechanisms to regulate their subcellular localization. Firstly, the PH domain confers recruitment of Gab proteins to plasma membrane patches enriched in specific phosphatidyl-inositol-phosphates (PIPs) [23-29]. In addition to the PH domain, Gab proteins use at least two additional mechanisms for their recruitment to activated plasma membrane-associated receptors. The first mechanism appears unique to the c-Met/Gab1 receptor/transducer system. Gab1 contains a specific c-Met binding domain (MBD), which encompasses aa residues 450 to 532 and confers the direct interaction between this docking protein and the c-MET RTK following its engagement by its ligand, hepatocyte growth factor (HGF) [17,30-32]. The MBD could be narrowed down to a sixteen amino acid motif (aa residues 486-501) called Met binding motif [31]. This direct interaction involves the activated kinase domain of c-MET and the MBD in Gab1 [17,31]. However, c-MET also recruits Gab1 *via *a second mechanism involving the small adaptor protein Grb2, and this represents the only mode of receptor interaction for Gab2 [33]. The significance of this indirect recruitment is underscored by the observation that a c-Met receptor mutant selectively defective in Grb2 binding fails to induce branching morphogenesis in the Madin-Darby canine kidney (MDCK) cell line model system [34] and by the non-viable phenotype of *knock-in *mice expressing a Grb2 binding-deficient Gab1 mutant [32]. This indirect mode of recruitment (Figs. 2 and 3) appears to apply to all other receptors recruiting Gab proteins. Phospho-tyrosine residues within the cytoplasmic tails of these receptors serve as docking sites for the SH2 and/or PTB domains of Grb2, which binds to the proline-rich regions in Gab1-3 via its C-terminal SH3 domain [11,33,35-40]. Shc proteins can serve as additional bridging adaptors between Grb2 and the tyrosine-phosphorylated receptors.

### Indirect recruitment of Gab proteins with the help of Grb2 adaptors

Vertebrate Gab proteins possess at least two regions that are potentially involved in the recruitment of Grb2 or other proteins containing SH3 domains such as Mona/Gads (Fig. 1; [11,41]). Such recruitment sites for SH3 domains were also identified in DOS and the Gab-like proteins identified in the sea squirt *Ciona *and the sea anemone *Nematostella *[42,43]. The small adaptor protein Grb2 contains a central SH2 domain flanked on each side by an SH3 domain [44]. Upon ligand binding, many cell surface receptors become tyrosine phosphorylated, which provides binding sites for the SH2 domain of Grb2 [3]. While being bound to the phosphorylated receptor, Grb2 can then use its two SH3 domains to recruit additional proteins to the activated receptor. For example, Grb2 binds to proline-rich stretches in the Ras-guanine nucleotide exchange factor SOS *via *its N-terminal SH3 domain, while it uses its C-terminal SH3 domain to bind to two SH3 binding motifs within Gab proteins [42,45].

Two recruitment motifs can be distinguished in Gab/Dos proteins, a "typical recruitment" motif and an "atypical" Grb2 binding site [46]. The typical Grb2 binding site, which occurs in Gab1/2/3, but not in SOC-1 and DOS, conforms to the canonical PXXP motif for SH3 domain binding [47]. In addition, both Gab1/2/3 as well as DOS and SOC-1 contain a so-called atypical Grb2 binding site with the recognition sequence PXXXR [46], which is also found in the SLP-76 and SLP-65/BLNK docking proteins [41,48]. Biochemical experiments by Lock *et al*. have demonstrated that both binding sites contribute to Grb2 binding [46] and consequently most functional studies addressing the Gab/Grb2 interaction utilize Gab mutants in which both recruitment motifs are mutated (ΔGrb2). However, these two sites may not be functionally equivalent. Using crystallography, peptide arrays and isothermal calorimetry, Harkiolaki *et al*. recently provided new insights into the interaction between the C-terminal SH3 domain of Grb2 and Gab2. In this study they demonstrated that both Grb2 binding sites contain the core consensus motif RxxK (with x for any amino acid) [42]. However, they also determined that the individual binding modes between the C-terminal SH3 domain of Grb2 and peptides derived from the typical and atypical Grb2 binding site differ significantly from each other. Consequently, this study provides a prime example of the flexibility of SH3 domains with regard to target recognition.

Since mutation/deletion experiments have clearly demonstrated the importance of the Gab2/Grb2 interaction for the activation of the various effector pathways controlled by this docking protein [39,40], important issues that remain to be resolved for many signalling systems are whether both recruitment sites are equally important, functionally redundant or are used in a stimulus-specific manner. In addition, it remains possible that the individual sites are used sequentially during the Gab recruitment process. Feller *et al*. (2002) have addressed the first issue for DOS by showing that mutation of either of the two Grb2 binding sites impairs R7 photoreceptor cell development in a moderate manner, while simultaneous mutation abrogates R7 development completely [43]. Furthermore, Yamasaki *et al*. (2003) have shown that the atypical Grb2 binding site plays a dominant role in the Gads/Grb2-mediated recruitment of Gab2 to the LAT signalling complex in the lipid rafts of T lymphocytes [41]. A final point of interest is that, although one might predict the Gab/Grb2 interaction to be constitutive, time course experiments have revealed that the Grb2/Gab ratio is increased by extra-cellular signals such EGF or IL-3 stimulation [8,9,39,49,50]. It remains to be tested as to whether this increase is caused by a conformational change of Gab that facilitates Grb2 binding or if this reflects indirect recruitment of this adaptor into the Gab2 signalosome by other proteins. Such a scenario could involve SHP2, which interacts with Grb2 as well [51].

A more complex indirect recruitment mechanism is utilized by Gab1 in FGF receptor signalling [14,52]. Here, the activated FGFR recruits first the docking proteins FRS2α/β, which are tethered to the plasma membrane by a myristylation anchor at their N-terminus and in addition contain a phosphotyrosine-binding (PTB) domain that mediates the direct interaction with FGF receptors [53]. Following interaction of FRS2α/β with the activated receptor, phosphorylated tyrosine residues within the FRS2 proteins serve as binding sites for the SH2 domain of Grb2 (Fig. 3). In turn, Grb2 binds with its C-terminal SH3 domain to Gab1, which is then recruited to the activated FGFR complex and becomes tyrosine phosphorylated. This mechanism plays an important role in FGF-induced PI3K activation, since this is mediated *via *Gab1/p85 interaction in the signalling complex [52]. It remains to be tested as to whether this mode of recruitment is also realized for Gab2 and Gab3 and for other receptor systems, such as NGF/TRK receptors, which also employ FRS2 proteins as signalling platforms [54]. The different strategies employed by particular cell surface receptors to recruit Gab docking proteins are summarized in Fig. 3.

### The Gab PH domain

Several PH domains are able to recognize specific phosphoinositides such as phosphatidylinositol-3,4,5-trisphosphate (PI_3,4,5_P_3 _or PIP_3 _in short), phosphatidylinositol-3,4-bisphosphate (PI_3,4_P_2_) as well as phosphatidylinositol-4,5-bisphosphate (PI_4,5_P_2_) [55,56]. Interestingly, Gab1/2 belong to the few proteins, which bind preferentially to the PI3K product PI_3,4,5_P_3_, which is only found within the plasma membrane, and less to PI_3,4_P_2 _and PI_4,5_P_2 _[23-25,40,57]. The PH domain plays an important role in the plasma membrane recruitment of Gab1 in cells stimulated *via *the EGF-, VEGF- or B cell antigen receptors (BCR) [23,26,27,58]. It is also required for recruitment of Gab1 to cell-cell contacts, and for the morphogenetic program triggered by the c-MET receptor [24,25]. In the case of Gab2, the PH domain mediates recruitment to phagocytic cups induced by FcγRI [28] and is required for bFGF-induced tyrosine phosphorylation of this docking protein in murine P19 teratocarcinoma cells [29]. In conclusion, these findings suggest that the PH domain might play an important role to localize or to concentrate Gab proteins to membrane areas where receptors are activated.

### Recruitment *via *the PH domain or adaptors - which mechanism predominates?

The PH domain mediated recruitment of Gab proteins provides the opportunity to modulate their membrane residency by PI3K and lipid phosphatases in a very dynamic manner without the need to disrupt large signalosomes such as the EGFR/Grb2/Gab complex that are potentially stabilised by various direct and indirect protein-protein interactions. A recent study by Sampaio *et al*. (2008) reiterates the importance of the PH domain by showing that it is required for the EGF-triggered recruitment of Gab1 to the plasma membrane in the presence of low doses of EGF, while the recruitment of this docking protein by high doses of EGF relies on Grb2 [59]. The dependency on the PH domain could be explained by the fact that, in the presence of low EGF concentrations, fewer EGFR molecules are auto-phosphorylated and thereby have a reduced potential to recruit binding partners such as the SH2 domain of Grb2. However, the reason(s) as to why high concentrations of EGF induced lower tyrosine phosphorylation of a Gab1 mutant impaired in Grb2 binding than a low concentration of this growth factor is unclear, but might be explained by competition with other PH domain containing proteins [59].

If the PH domain were to play such an important role under low growth factor stimulation, one would expect that the membrane recruitment mechanisms reliant on protein-protein interactions such as the c-MET/Gab1 and Grb2/Gab interactions would be largely dispensable. In the following, we review several lines of evidence from various experimental settings indicating that the PH domain alone cannot confer long-term plasma-membrane residency or ensure adequate physiological Gab signalling. For example, the MBD plays an important role in Gab1 recruitment under certain circumstances [33]. A strong interaction with particular activated receptors is mediated *via *the Grb2 binding sites, as indicated by various lines of evidence. Firstly, the tyrosine phosphorylation of Gab1 is drastically reduced in mouse embryonic fibroblasts (MEFs) lacking Grb2 or expressing a functionally impaired Grb2 protein in which its SH2 domain has been rendered non-functional by the E89K *knock-in *mutation [60]. The opposite experiment in which the Grb2 binding sites in Gab1 were mutated also resulted in an impaired tyrosine phosphorylation of Gab1 [46,59,61,62]. A similarly impaired tyrosine phosphorylation of Gab1 was observed in Fr3T3 cells expressing a Grb2 binding deficient and transformation impaired mutant of the Tpr-Met oncoprotein [63]. Most importantly, despite the presence of intact PH and MET-binding domains, *knock-in *mice that express a Gab1 mutant lacking the Grb2 binding sites display an embryonic lethal phenotype and defects in liver, placenta and craniofacial development [32]. This finding underscores the importance of the Gab1/Grb2 interaction. Furthermore, a Gab2 mutant lacking both typical and atypical Grb2 binding sites displays a reduced and short-lived tyrosine phosphorylation in EGF-stimulated human mammary epithelial cells and in Fcε RI-stimulated murine bone marrow derived mast cells (BMMCs) [39,40]. This suggests that the Grb2 binding sites, while not essential to achieve a certain degree of tyrosine phosphorylation, are necessary to sustain tyrosine phosphorylation, in particular at time points at which PI3K levels have already returned to baseline levels due to the action of PIP_3 _hydrolysing phosphatases such as SHIP and PTEN [64,65]. This notion is further supported by the plethora of receptors employing Grb2 as a recruitment device for Gab proteins (Fig. 3).

Overall, it appears that the relative roles played by these alternative recruitment mechanisms are context-dependent. The reports reviewed in this section invite for detailed future studies that not only take the amount and timing of the extra-cellular stimulus into account, but also consider the lineage and transformation status of the cell lines. Indeed, the Gab/Grb2 interaction might be more relevant in primary tissues or immortalized cell lines such as BMMCs and MCF-10A, than in particular tumour cell lines often used in signalling studies, e.g. Jurkat or MCF-7 cells, which display elevated PIP_3 _levels due to the loss of PTEN expression or *PIK3CA *mutations, respectively [65-68]. Lastly, it should be noted that even in the same cellular setting, particular Gab proteins may differ in their requirement for PH domain-mediated plasma membrane recruitment. For example, van den Akker *et al*. showed that the EPO-induced tyrosine phosphorylation of Gab2 is much more reliant on PI3K activity than that of Gab1 [69].

## Positive regulation of Gab proteins and downstream effectors

As *bona fide *signal transducers, Gab proteins not only possess structural motifs for their receptor recruitment, but also contain features that are involved in the transduction, localization and amplification of receptor-derived signals (Fig. 1). At the moment, the SHP2/Ras and the PI-3K/AKT pathways are considered as the two major effector arms of Gab proteins. However, a series of biochemical and genetic studies as well as yeast-two-hybrid (Y2H) screens have identified additional Gab effector proteins such as PLCγ-isoforms [8,70], adaptor proteins of the Shc [9,61,71] and Crk families [72-76], the lipid phosphatase SHIP [71], the Ras-GTPase activating protein RasGAP [77], GC-GAP [78] and transcriptional activators STAT3 and STAT5 [50,79-81]. In the following sections we will provide an update regarding recent insights into these effector pathways.

### Tyrosine phosphorylation of Gab proteins

A fundamental mechanism for regulation of Gab-mediated signal transduction is site-specific tyrosine phosphorylation of these docking proteins. Depending on the particular Gab family member, tyrosine phosphorylation may provide recruitment sites for the SH2 domains of the tyrosine phosphatase SHP2, adaptors of the Crk family, PLCγ and the regulatory subunit of PI3K, p85 [11,12]. However, the kinases and phosphatases controlling the phosphorylation status of these tyrosine residues are in many cases still ill-defined. While, at least *in vitro*, RTKs such as the EGFR are able to phosphorylate Gab1 directly [82], it is becoming increasingly evident that a variety of systems such as RTKs, antigen receptors, cytokine receptors and even the Bcr-Abl oncoprotein "sub-contract" PTKs of the Src-, Syk/ZAP-70 and JAK families to drive the tyrosine phosphorylation of Gab1/2 [37,83-94]. In some instances, a cascade of PTKs regulates Gab phosphorylation, such as the Bcr-Abl/JAK2/Lyn pathway in human CML cells [91,95]. Also, it is possible that individual PTKs might target distinct tyrosine residues in Gab proteins. By recruiting various effectors with SH2 domains, Gab proteins mediate not only signal amplification, but, as a function of the recruitment of distinct enzymatic activities, also channel the receptor-derived signals into pathways with distinct biological properties (Fig. 2). Thus, Gab proteins act as a nucleation core of an entire signalling subsystem, which we will dissect in the following sections.

### The SHP2/Ras/ERK pathway

Probably the best-characterized effector arm of Gab proteins is mediated *via *the protein tyrosine phosphatase SHP2. SHP2 contains tandem SH2 domains, the most N-terminal of which confers auto-inhibition of the C-terminal phosphatase domain [96,97]. Many SHP2 interaction partners including the Gab proteins contain two SHP2 binding sites, which, if phosphorylated, will act as a bi-phosphoryl tyrosine activation motif (BTAM) and confer simultaneous binding of both SH2 domains, thereby relieving auto-inhibition [96,98]. Thus, SHP2 interaction partners like Gab proteins might not only act as recruitment platforms, but also as allosteric activators. But what are the functional consequences of Gab-mediated SHP2 recruitment and activation? The best understood effect mediated by the Gab/SHP2 interaction is the sustained and/or increased activation of the ERK/MAPK pathway (Fig. 2). This effect occurs in response to a variety of stimuli, including treatment of cells with EGF, VEGF, HGF and LPA [35,39,48,62,98-101]. However, in certain cellular contexts, the Gab/SHP2 complex also positively regulates other downstream pathways. These include c-Kit-induced Rac activation [102] as well as β1-integrin- and growth factor-induced PI3K activation [39,103]. The detailed mechanisms involved in Gab/SHP2-mediated regulation of Rac and PI3K have yet to be resolved. In cultured mammalian cells, recruitment of SHP2 to particular Gab proteins regulates diverse biological endpoints, including PDGF-induced cytoskeletal organization and VEGF-induced migration in endothelial cells [62,104], cell adhesion and migration of Ba/F3 haematopoietic cells [103], epithelial morphogenesis in MDCK cells [99] and acinar growth of MCF-10A mammary epithelial cells [39]. These studies have been complemented by epistasis analyses in *Drosophila melanogaster *and *Caenorhabditis elegans *[7,21,105]. Recently, the physiological significance of the Gab/SHP2 interaction for Ras/ERK activation was underscored by the generation of *knock-in *mice expressing a Gab1 mutant lacking the tyrosine residues involved in SHP2 recruitment (Gab1^ΔSHP2^). These mice display impaired development of muscles and the placenta [32], an organ known to be extremely sensitive towards aberrant ERK signalling [106].

Taken together, these findings demonstrate that SHP2 is an important positive modulator of ERK activation. However, why the recruitment of SHP2 by Gab proteins is required for full activation of ERK signalling downstream of receptor tyrosine kinases is still not fully understood, but several mechanisms may contribute. Firstly, SHP2 de-phosphorylates binding sites for p120^Ras-GAP ^on the activated receptors for PDGF and EGF [107,108] and also on Gab1 [77] and thereby counteracts Ras inactivation. In the latter case, p120^Ras-GAP ^is recruited *via *its SH2 domain to phosphorylated Y317 on Gab1, which is dephosphorylated by SHP2. Like Gab1, DOS is also de-phosphorylated by the SHP2 orthologue Corkscrew (CSW) resulting in enhanced Ras activation [6]. Secondly, Shp2 dephosphorylates recruitment sites for the Src-inactivating kinase Csk on the transmembrane glycoprotein PAG/Cbp [109] and paxillin [110], leading to enhanced activity of Src family kinases. These data are consistent with a report showing that the expression of a fusion protein consisting of the Gab1 PH domain and SHP2 does not only induce constitutive ERK pathway activation, but also enhances activation of Src [100].

### The PI3K effector arm

Through the recruitment of PI3K to activated receptors, Gab proteins contribute to the initiation of signalling pathways promoting cellular growth, survival, migration and proliferation [11]. The generation of *knock-in *mice expressing a Gab1 protein defective in p85 recruitment (Gab1^Δp85^) demonstrated that the interaction between Gab1 and PI3K downstream of the EGFR is important in embryonic development for eyelid closure and for keratinocyte migration [32]. Nevertheless, their viable phenotype also indicates that the Gab1/p85 interaction is, in comparison to the interactions of Gab1 with c-Met, Grb2 and SHP2, a relatively dispensable interaction during mouse development. While a Gab1 gene *knock-out *is embryonic lethal [61,111], Gab2 deficient mice are viable [112-114]. However, the essential role of Gab2 in IgE-mediated allergic responses is attributed to its function in coupling FcεRI to PI3K activation [112]. The role of Gab2 in FcγR-mediated phagocytosis also seems to be dependent on the recruitment of PI3K [11]. Some receptors recruit PI3K both *via *direct p85 binding sites and *via *Gab proteins, for example c-Kit and the NGF receptor [63,115]. A recent study showed that a splice variant of c-Kit that recruits Gab2 induces much stronger activation of the PI3K pathway than an isoform that does not bind Gab2 and recruits PI3K only directly [116]. Similarly, the B and T cell antigen receptors recruit PI3K *via *co-receptors and *via *Gab2 [27,117]. Thus, Gab proteins serve as amplifiers of PI3K signalling in many receptor systems, in particular for those lacking direct p85 binding sites such as the IL-3 receptor. This receptor activates PI3K *via *a Shc/Grb2/Gab2 complex and other cytokine receptors lacking direct PI3K binding sites might use the same pathway [38]. Lastly, it should be emphasised that Gab1-induced PI3K activation can amplify receptor signalling by generating a positive-feedback loop, as described for the EGFR system by Rodrigues *et al*. (2000) [23].

### Gab signalling to PLCγ

Association of Gab proteins and PLCγ 1/2 most likely reflects a direct interaction involving tyrosine-phosphorylated residues on the docking protein and the SH2 domains of the PLCγ isoform. For example, HGF induces tyrosine phosphorylation of Gab1 at Y307, Y373 and Y407, which in turn recruit PLCγ1, a critical event for MET-induced branching morphogenesis of MDCK cells [118]. Gab2 also interacts with PLCγ2 in FcεRI-stimulated RBL-2H3 basophilic leukemia cells and RANKL-stimulated primary osteoclasts [70,119]. Interestingly, Mao *et al*. (2006) found that PLCγ2 not only interacts with Gab2, but also enhances its interaction with the receptor RANK and its tyrosine phosphorylation, suggesting that PLCγ2 plays a scaffolding or recruitment role in the RANK/Gab2 relationship [70]. The physiological relevance of the RANK/PLCγ2/Gab2 axis is supported by the observation that mice deficient in RANKL, RANK, PLCγ2 or Gab2 develop an osteopetrotic phenotype (see below). However, while these studies identify particular PLCγ isoforms as important effectors or regulators of mammalian Gab proteins, a DOS protein lacking the putative PLCγ binding sites is able to rescue the phenotype of DOS-deficient flies [105], indicating that the DOS/PLCγ interaction does not play an essential role in this context.

### Shc proteins - just companions of Grb2?

Another prominent component of immuno-purified Gab signalling complexes are the Shc adaptor proteins. In many cases, however, it is still unclear as to whether Shc interacts directly with Gab proteins or is recruited *via *Grb2. The latter mechanism has been demonstrated for Gab2 signalling complexes from EGF-stimulated mammary epithelial cells and from Fcε RI- or stem cell factor (SCF)-stimulated mast cells [39,40,49,102]. Similarly, Liu *et al*. (2001) identified Shc in Gab2 complexes from M-CSF stimulated cells, but failed to purify Gab2 using GST fusion proteins bearing either the SH2 or PTB domain of Shc [120]. These data argue against a direct interaction. However, it should be noted that the Scansite program [121] predicts putative binding sites in Gab1 and Gab2 for the SH2 domain of Shc and Far Western blot analyses have demonstrated a direct interaction between the GST-Shc SH2 domain and tyrosine phosphorylated Gab1 purified from BCR-stimulated B cells [122]. Consequently, Shc proteins might be able to interact with Gab proteins under certain circumstances. Be that as it may, the exact role of Shc proteins in the Gab signalosomes is still not completely resolved. Do they only serve as "bridging molecules" (Fig. 2 and 3) or do they fulfil additional functions, e.g. by concentrating additional regulators of Gab signalosome components such as 14-3-3 proteins [123] or the SHIP lipid phosphatases [124,125]? Indeed, SHIP1 and 2 have been found in Gab signalosomes in a variety of settings, e.g. in Gab1 complexes purified from B cells stimulated either through the BCR alone or in co-clustering experiments involving both BCR and the inhibitory FcγRIIb [124,125]. Similarly, SHIPs have also been detected in Gab1/2 signalosomes isolated from EPO-stimulated UT-7 cells [71], a human pluripotent leukemia cell line, in FcεRI-stimulated RBL-2H3 cells [126] and in M-CSF-stimulated FDCP1 cells, that represent mouse myeloid progenitors [120]. Although a direct interaction between the SH2 domain of SHIP1 and Gab2 was demonstrated in Far-Western blot experiments [120], several studies suggest that these interactions are indirect and mediated *via *Shc [124,125]. The role of SHIPs in Gab signalling complexes is still ill-defined, however, an attractive idea is that they counteract the contribution of the Gab proteins to local PI3K signalling.

### The role of Gab proteins in the activation of small GTPases

Crk proteins constitute another group of Gab interaction partners. These adaptor proteins consist of one N-terminal SH2 domain followed by one (CrkI) or two (CrkII) SH3 domains [127,128]. As shown in Fig. 1, both Gab1 and Gab2 as well as DOS contain multiple consensus binding sites (Y-X-X-P) for the SH2 domain of Crk proteins [127,129]. The interaction of Gab proteins with these adaptors has been observed in a variety of cell types and downstream of distinct receptor/transducer systems such as RTKs, antigen and certain cytokine receptors [74,127,129]. In turn, Crk proteins recruit particular effectors *via *their SH3 domains e.g. guanine nucleotide exchange factors for Rac and Rap-GTPases. Thereby, they potentially regulate cellular motility, adhesion and morphology [74,129]. Interestingly, Watanabe *et al*. have recently demonstrated that HGF/c-MET signalling in human synovial sarcoma cell lines induces sustained tyrosine phosphorylation of Y307 on Gab1, which serves as a recruitment site for both Crk and PLCγ [129,130]. The recruitment of Crk to this residue is not only pivotal for downstream signalling events, e.g. Rac activation, and enhanced cell scattering, invasive behaviour and xenograft growth, but is also required for the sustained tyrosine phosphorylation of Gab1 itself [129]. In a follow-up study, the same group demonstrated that Y307 is phosphorylated by Src. This enhances cellular migration and contributes to the membrane recruitment of Gab1 in HGF-stimulated MDCK cells and the organisation of focal adhesion complexes [93]. Furthermore, other studies have shown that formation of the Gab1/Crk complex is a critical event in c-Met induced activation of the JNK pathway, an event downstream of Rac activation and a prerequisite for several of the aforementioned morphological changes and efficient cellular transformation [72,131,132]. Interestingly, a recent report has demonstrated that the p85 and Crk binding sites in Gab1 play a pivotal role in the c-Met mediated entry of the intracellular bacterium *Listeria monocytogenes*, implicating the Gab1/Crk complex in promotion of cytoskeletal rearrangements required for pathogen internalization [133].

In a Y2H screen conducted with a portion of Gab2 (aa 120-587) as a bait, Zhao *et al*. (2003) isolated a novel GTPase activating protein (GAP) for Rho-GTPases, which was named GC-GAP [78]. This interaction was subsequently confirmed by co-immunoprecipitation experiments. GC-GAP is highly expressed in brain and displays *in vitro *GTPase stimulating activity towards RhoA, Rac1 and Cdc42 and towards Rac1 and Cdc42 upon ectopic expression in HEK293T cells. RNAi-mediated suppression of GC-GAP was correlated with reduced proliferation of C6 astroglioma cells. Although the original identification of the Gab2/GC-GAP interaction in the Y2H screen suggests a direct interaction between both proteins, it should be noted that GC-GAP also interacts in mammalian cells with the N-terminal SH3 domain of Crk [78].

A recent study by Paliouras *et al*. (2009) has identified the Ser/Thr-kinase and Rac/Cdc42 effector PAK4 as a specific interaction partner of the Gab1 isoform [130]. PAK4 binds in a phosphorylation-dependent manner *via *its GEF-interacting domain to a region in Gab1 located between the PH domain and the first of the three Crk binding sites (aa 116-234). Interestingly, the authors could show that ectopically co-expressed Gab1 and PAK4 cooperate in HGF-induced epithelial cell scattering and invasiveness and that PAK4 knockdown or deletion of the PAK4 recruitment region impaired these biological responses.

### The Jak/STAT pathway

Although the molecular details remain ill-defined, the Gab2 isoform is increasingly implicated in JAK/STAT signalling. An early study demonstrated that in CD4-positive T cells derived from the rare human neoplasia *mycosis fungoides*, tyrosine-phosphorylated Gab2 interacted with SHP2 and STAT5a in a IL-2- regulated fashion [79]. Later, Arnaud *et al*. (2004) uncovered a complex interplay between Gab2, SHP2 and STAT5 in IL-2 stimulated T cells [80]. Here, S623 becomes phosphorylated in a negative feedback loop by activated ERK, which in turn reduces the potential of Gab2 to interact with SHP2 *via *the phosphorylated tyrosine residues Y614 and Y643. Interestingly, activation of the ERK pathway was blunted, as expected by other studies, by the Y614F mutation and slightly increased by the Gab2^S623A ^mutant. In contrast, IL-2-induced STAT5 activation was enhanced by the SHP2 binding mutant Gab2^Y614F ^and inhibited by Gab2^S623A^. These data indicate a potential role of STAT5, its interaction partners or its upstream kinases as SHP2 substrates.

Additional observations support the concept of a functional cooperation between STAT5 and Gab2. First, the murine *gab2 *gene is one of the top candidates on the modifier locus located on chromosome 7 that modulates the engraftment of hematopoietic stem cells (HSC) during steady-state haematopoiesis, a process dependent on intact cytokine signalling [134]. Second, two studies from the Gouilleux laboratory have shown that constitutively active mutants of STAT5 (caSTAT5) not only associate with Gab2, but also require this docking protein for the efficient induction of Ba/F3 cell proliferation *via *the Ras/ERK and PI-3K/AKT pathways [50,135]. In this system, caSTAT5-induced cell proliferation, as well as ERK and Akt activation, is dependent on Gab2/p85 binding. Interestingly, the authors also demonstrate that the basal tyrosine phosphorylation of Gab2 is increased in caSTAT5-expressing Ba/F3 cells [50]. This suggests that PTKs are recruited to the Gab2 signalosome by caSTAT5 or that STAT5 protects Gab2 against dephosphorylation by PTPs, e.g. Shp2. In support of the latter model, Gab2 is not associated with Shp2 in caSTAT5 expressing cells. However, it remains unclear at present whether the Gab2/STAT5 interaction is mediated *via *a direct interaction or *via *a mutual binding partner such as p85. Clearly, further work is required to characterize mechanisms underpinning the interplay between Gab2 and STAT5, and to determine how STAT5 antagonizes Shp2 recruitment to this docking protein.

In addition to the STAT5/Gab2 relationship, Ni *et al*. (2007) have demonstrated that murine and human Gab2 orthologues, but not Gab1, contains a canonical STAT3 binding motif (YXXQ). Using a Y194F substitution mutant, the authors could demonstrate that this site is indeed required for the recruitment of STAT3 and the efficient Friend erythroleukemia virus-mediated transformation of murine hematopoietic progenitors [136]. It remains to be seen as to whether this site is also involved under more physiological circumstances and in the recruitment of other STAT proteins such as STAT5.

## Gab proteins are ancient elements of the metazoan signalling toolbox

**Figure 4:**
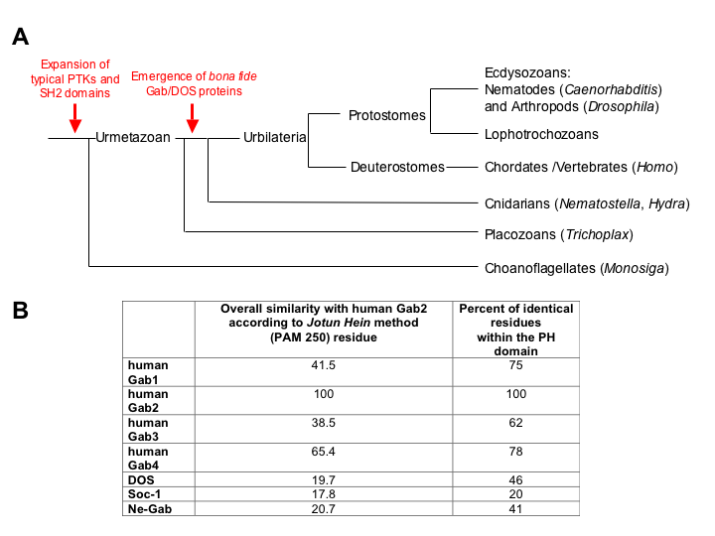
**(A) Simplified phylogenetic tree illustrating the emergence of PTK signaling systems and the emergence of Gab/Dos proteins**. The tree is based on recent insights into metazoan evolution and highlights the position of the model organisms in which *bona fide *Gab/Dos proteins have been identified [137,139,140,240,241]. The distances between the individual clades do not represent phylogenetic or temporal distances. **(B) Overall sequence similarity and sequence identity across the PH domains of the various Gab/DOS proteins discussed in this review**.

Recent genome analyses have revealed that the emergence of PTK signalling networks precedes the advent of true multi-cellularity and that these expand dramatically at the base of the animal kingdom (Fig. 4A; [137-140]). Consequently, these analyses should aid in the identification of the time point of the emergence of Gab, DOS and SOC proteins and would assist in the design and interpretation of structure-function analyses of Gab proteins. We reasoned that if the hallmarks of a Gab protein were the presence of an N-terminal PH domain followed by Pro-rich sequences enabling the recruitment of SH3 domains and the presence of multiple tyrosine phosphorylation motifs for the recruitment of SH2 domains, then it would be possible to search for Gab proteins in lower metazoans and to identify proteins that resemble the last common ancestor of the DOS, SOC and Gab proteins. To this end, we made use of the recently published genomes of the choanoflagellate *Monosiga brevicollis*, which represents an outgroup to metazoans, and the basal metazoan *Trichoplax adhaerens *and the starlet sea anemone *Nematostella vectensis*. Whilst there is no evidence for *bona fide *Gab/DOS proteins in either the *Monosiga *nor *Trichoplax *genomes (our own observations), we and the Feller group identified a Gab-like protein in the *Nematostella *genome (GenBank entry XP_001636529; [42]). This conceptual protein (NeGab; Fig. 1) carries an N-terminal PH domain of the Gab type (PH_GAB) followed by potential recruitment sites for SH3 domains, of which two and three align almost perfectly with the typical and atypical Grb2 binding domains of human Gab proteins, respectively. These motifs also contain the key residues for the interaction with the C-terminal SH3 domain of Grb2 [42]. It should be noted that the typical Grb2 binding site does not occur in the various DOS proteins and SOC-1 [20]. Importantly, our Scansite analysis revealed that NeGab also carries several tyrosine residues that match phosphorylation motifs involved in the recruitment of SH2-containing proteins such as the Gab signalosome components p85, CrkL and PLCγ (Fig. 1). Overall, these findings are consistent with the presence of orthologues of the Gab signalosome components Grb2, Shc, PLCγ and Crk in *Nematostella *[137] and database entries for SHP2- and p85-like proteins for another Cnidarian, *Hydra magnipapillata *(our unpublished observations). However, it should be noted that, whilst there are also tyrosine residues in NeGab that align with those involved in the recruitment of SHP2 to mammalian Gab proteins, the surrounding residues are not conserved and therefore do not constitute a *bona fide *recognition motif for both SH2 domains of mammalian SHP2 [141]). These findings suggest that either NeGab does not recruit the corresponding SHP2 orthologue or that the NeGab/SHP2 interaction takes place by other means. Nevertheless, according to a Scansite prediction [121], the SH2 binding motifs in NeGab align with one of the CrkL (Y266 in human Gab2) and p85 (Y584) recruitment sites in mammalian Gab proteins. Taken together, these data suggest that at least the Gab/Crk, Gab/PLCγ and Gab/PI3K connections were already established in the precambrium and that a Gab-like docking protein was an early feature of the metazoan PTK signalling toolkit (Fig. 4A).

So far, all sequenced genomes of invertebrate model organisms including those closer to the basis of vertebrates such as the chordates *Ciona *and *Branchiostoma *possess only one Gab-like protein [42,142]. This suggests that the three Gab paralogues were generated by the genome-wide duplications that occurred with the emergence of vertebrates and have diversified subsequently [42,143]. However, the Gab family appears to expand further in mammals. An enigmatic entry in genomic databases represents the *GAB4 *gene, for which entries at both the genomic DNA and transcript level have been recorded for humans and chimpanzees. The human *GAB4 *gene is located on chromosome 22q11.1 and its nucleotide sequence is most related to Gab2 (65% similarity: Fig. 4B), which is encoded on chromosome 11q13. *GAB4 *contains a *bona fide *exon/intron structure suggesting that this gene is not a retro-transposon-like element that has been derived from a Gab2 transcript. Furthermore, the detection of Gab4 ESTs in testicular tissue, as well as sequence differences between Gab2 and Gab4, suggest that this gene is indeed actively transcribed and might give rise to a functional Gab4 protein [144]. While the putative Gab4 contains potential binding sites for SHP2, only one of the three p85 binding sites is conserved and it lacks the typical Grb2 binding site (Fig. 1). The expression pattern, signalling mechanisms and functional roles of the Gab4 protein remain to be characterized.

## Physiological functions of Gab proteins

Various analyses have shown that most mammalian cell types express more than one Gab family member [19], suggesting that the individual proteins are not functionally redundant. This hypothesis is supported by the phenotypes of Gab1-3 gene *knock-out *mice. While Gab2 and Gab3 *knock-out *mice are viable and have a normal life span [112,115,145], Gab1 deficiency causes embryonic lethality due to severe defects in heart, placenta, liver, skin and muscle development [61,111]. In line with the specific and intimate relationship between Gab1 and c-Met, it is perhaps not surprising that mice lacking Gab1 phenocopy many of the aspects of HGF- and c-MET-deficient mice, such as early embryonic lethality owing to placental defects, reduced liver size and defects in the migration of muscle precursor cells [32,61]. As already discussed above, the generation of *knock-in*-mice carrying mutations in either the SHP2 or the p85 binding sites of Gab1 revealed that these interactions play distinct roles in embryonic development [32]. Interestingly, enforced membrane localization of Gab2 through the addition of a myristoylation signal together with the introduction of the MBD from Gab1 is sufficient to confer a Gab1-like behaviour to Gab2 in HGF-stimulated MDCK cells [146]. These findings indicate that in the case of Gab1 and Gab2, differences in their subcellular compartmentalization, rather than in coupling to effectors, leads to distinct biological properties.

Using cardiomyocyte-specific Gab1/Gab2 double-deficient mice, Nakaoka *et al*. (2007) could show that both Gab1 and Gab2 play an important role in the postnatal maintenance of cardiac function [147]. Neuregulin-1β (NRG1 β), a paracrine factor produced from endothelium and a major ligand for the ErbB2/ErbB3 heterodimer, induces marked tyrosine phosphorylation of Gab1 and Gab2 leading to activation of ERK and AKT and the up-regulation of angiopoietin 1 in the heart. These responses were absent in Gab1/2-double deficient mice, which exhibited high postnatal mortality and various signs of cardiac insufficiency.

Gab2 plays an important albeit not essential role in the development of various haematopoietic lineages [113], except for NK cells [148]. Resulting defects in Gab2-deficient mice can be attributed to the reduced responsiveness of hematopoietic progenitors to early-acting cytokines [113]. Importantly, Gab2-deficient mast cells display a drastic phenotype. They fail to degranulate and to secrete cytokines following activation of the FcεRI antigen receptor [112]. Consequently, allergic reactions including systemic anaphylaxis are markedly impaired in Gab2-/- mice. These mast cell activation defects reflect the pivotal role of Gab2 as an amplifier for FcεRI induced PI3K activation. Similarly, knockdown of Gab2 expression with siRNA or antisense oligonucleotides in RBL-2H3 rat basophilic leukaemia cells, a widely used model system for mast cells, results in drastically impaired degranulation and cytokine production [149,150]. Furthermore, murine mast cell development is impaired, because of weakened c-Kit signalling [115]. These findings suggest that Gab2, which is often up-regulated in inflammatory diseases [151], might be an important target for novel therapies against inflammation and allergy. However, both Gab1 and Gab2 are involved in the aggregation of platelets triggered by the collagen receptor GPVI [152]. Thus, there might be a certain degree of redundancy between Gab1 and Gab2 for some functions within the hematopoietic system, which could be dissected further using inter-crosses between Gab2-deficient mice and a conditional Gab1 *knock-out*.

Gab2-deficient mice also display an osteopetrotic phenotype that is explained by the role of Gab2 as a key regulator of RANK signalling [114]. Bone homeostasis is the result of an intricate balance between the anabolic action of mesenchymal osteoblasts and the catabolic action of osteoclasts, which represent a specialized type of monocyte/macrophage lineage. In agreement with its pivotal role in the differentiation of various haematopoietic lineages [113,115], Gab2 deficiency results in defective osteoclast differentiation, which causes decreased bone resorption and a subsequent systemic increase in bone mass. Gab2 is tyrosine-phosphorylated in RANK ligand stimulated osteoclast progenitors and it interacts with the C-terminal domain of RANK. These reports are complemented by a study showing that Gab2 plays distinct roles in osteoclastogenesis in different phases of skeletal development. According to this study, Gab2-deficient mice display enhanced bone formation at six weeks of age and reduced osteoclast differentiation at twelve weeks of age [153]. In addition to the RANK signalling pathway, EGFR signalling has also been implicated in osteoclast differentiation. Since Gab2 functions as signal transducer in both pathways, it has been suggested that the crosstalk between the two receptors might be mediated by Gab2. Indeed, an interaction of the EGFR, RANK and Gab2 could be shown. Moreover, stimulation of osteoclasts with RANKL induces tyrosine phosphorylation of the EGFR implying that the EGFR is transactivated by RANK [154]. Recently, the PTK Lyn has been shown to be recruited to the RANK/Gab2 signalling complex and to act as a negative regulator of osteoclast differentiation by inhibiting the tyrosine phosphorylation of Gab2 [85]. This mechanism involves Lyn-mediated phosphorylation of the tyrosine phosphatase SHP-1. As a consequence, Lyn-deficient mice display bone loss due to increased osteoclastogenesis. Therefore, Lyn can either enhance [86,88,95] or attenuate [85] Gab2 tyrosine phosphorylation, depending on cellular context. These findings further illustrate how fragmentary our knowledge still is in respect to the mechanisms regulating Gab phosphorylation.

All three mammalian Gab proteins are expressed in neuronal tissues [10,19,29,155], however their precise role in the CNS remains to be elucidated. Several reports suggest that the individual Gab proteins exert important and potentially non-redundant roles in the nervous system. Firstly, Korhonen *et al*. (1999) reported that ectopic expression of Gab2 in PC12 cells increased NGF-independent neuronal differentiation and survival *via *PI3K- and MEK-dependent pathways [155]. Similarly, Gab2, but not Gab3 acts downstream of FGF receptors in order to ensure the survival of various stem cell models during retinoic acid-induced neuronal differentiation [29]. Interestingly, this study also demonstrated that the expression of Gab2 is strongly up-regulated during neuronal differentiation and that Gab2 requires its PH domain and p85 recruitment sites to confer bFGF-mediated survival.

So far, studies have not identified any specific roles for Gab3. Genetically-modified mice deficient for Gab3 are healthy and viable and despite the strong up-regulation of this protein during macrophage development, no obvious phenotype was identified in Gab3-deficient macrophages [145].

## Negative regulation

Gab proteins fulfill critical roles in the communication between various receptor classes and several signalling pathways involved in the control of proliferation, cell death, migration and differentiation. Consequently, their expression, subcellular localisation and signalling competent state must be strictly regulated. Although our knowledge about these processes is still very limited, it is becoming apparent that several layers of negative regulation are applied to Gab docking proteins, which we will now discuss.

### Negative regulation by phosphatases

Firstly, as the PH domain plays an important role in membrane recruitment, Gab signalling is influenced by the balance between the activities of PI3K and lipid phosphatases such as PTEN or SHIP1/2. As discussed above, the latter proteins are recruited into Gab signalosomes [156]. Similarly, "PH domain-only" proteins such as the recently-described p53 target and putative tumour suppressor gene product PHLDA3 may negatively influence the membrane recruitment of Gab proteins through direct competition for PI3K products [157]. Indeed, such a scenario is supported by experiments in which the expression of the isolated PH domain of Gab1 suppressed EGF-induced ERK and AKT activation in breast cancer cell lines [158].

Secondly, tyrosine-phosphorylated Gab docking proteins recruit SHP2 and it is therefore highly likely that the phosphorylation of certain tyrosine residues and their associated downstream signalling events are directly regulated by this protein tyrosine phosphatase (PTP). Indeed, studies on both DOS and Gab1 have shown that they are dephosphorylated by CSW and SHP2, respectively [6,21,159]. Furthermore, the tyrosine residues implicated in the recruitment of p85 and RasGAP to Gab1 are substrates of SHP2 [77,160], which could explain as to why SHP2 mutants with impaired phosphatase activity promote the interaction between Gab1 and a GST-p85 fusion protein [161]. Although this has not been proven so far, it is conceivable that a comparable mechanism can be applied to the Gab2 signalling complex and that the presence of SHP2 in the Gab2 signalosome controls p85 recruitment and the extent of PI3K signalling. Indeed, such a scenario might explain as to why SHP2 recruitment dominates over p85 recruitment in the early phase of EGF-induced Gab2 activation [39] and, given the reports that p85 is accompanied by STAT5 into the signalosomes [135], why Shp2 recruitment is inversely correlated with STAT5 binding [80].

### Regulation of Gab protein expression

A third regulatory layer is the control of the expression level of Gab proteins by various mechanisms acting at the transcriptional and post-transcriptional levels. Although the regulation of *GAB *gene promoters remains poorly-characterized, one study has shown that transcription of the *GAB*2 gene is induced by the transcription factor E2F [162]. Furthermore, Gab2 expression is estrogen-regulated in hormone-responsive breast cancer cells [163] and studies in various cellular systems have revealed that Gab2 and Gab3 are up-regulated during cellular differentiation processes [19,29,145,164].

Another study has demonstrated that Gab2 is subject to ubiquitin-mediated degradation in FcεRI-stimulated RBL-2H3 basophilic leukaemia cells [165]. However, it remains to be seen as to whether this mode of negative regulation can be extended to other signalling systems and cell types.

### The hidden layer of complexity - fine tuning of docking proteins by Ser/Thr-phosphorylation

**Figure 5:**
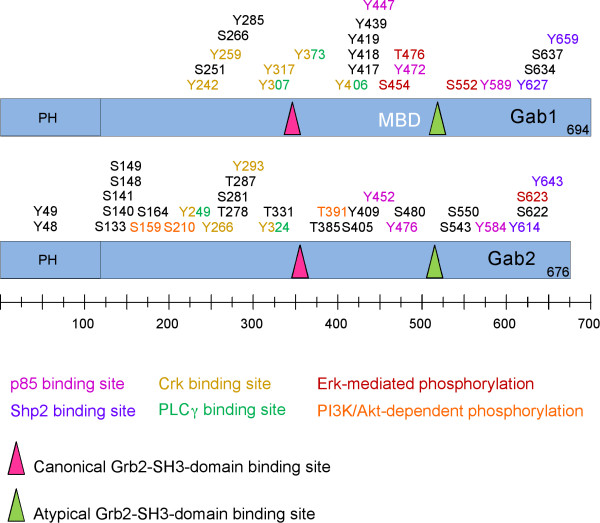
**The Phosphomaps of human Gab1 and Gab2**. This cartoon is based on information from sources cited in the main text and on the Phosphosite homepage http://www.phosphosite.org. Phosphotyrosine residues involved or strongly implicated in the recruitment of the SH2 domain containing effectors Shp2, p85, Crk and PLCγ are indicated. Ser/Thr-residues phosphorylated in an ERK- or PI3K-dependent manner are also shown.

A fourth and emerging mode of negative regulation of docking proteins is mediated by Ser/Thr-phosphorylation, which is often correlated with their reduced tyrosine phosphorylation and/or changes in subcellular localisation (see Refs. [15,97,166] for review). Indeed, early in Gab signalling research, the dramatic electrophoretic mobility shift displayed by these docking proteins upon growth factor or cytokine stimulation was attributed to phosphorylation events, although the sites and signalling pathways remained largely ill-defined until the recent advent of sensitive phospho-proteomics. Since then there is accumulating evidence that many docking proteins including those of the Gab family are targeted by several immediate early feedback loops involving various classes of protein Ser/Thr-kinases (Fig. 5; for review see Refs. [97,166,167]). Bioinformatic analyses, e.g. using the NetPhos 2.0 algorithm, predict that Gab1 and Gab2 contain 47 and 76 potential Ser/Thr-phosphorylation sites, respectively [8,168]; our unpublished observations). Indeed, recent phospho-proteomic analyses on Gab2 and SLP-65 [49,169] have underscored our concept that docking proteins are heavily phosphorylated in a dynamic manner and thereby act as the centre of entire signalling subsystems or hubs [170] as it is also depicted in Fig. 2. In the following section we will provide an overview of how this field has progressed over the last five years.

### ERK mediated feedback phosphorylation of Gab1

The first reports on the feedback phosphorylation on Ser/Thr-residues of Gab1 by the MAPK ERK were reported about ten years ago by the Cantley group and were subsequently confirmed by others in a variety of experimental settings [90,168,171,172]. Six ERK-dependent phosphorylation sites (T312, S381, S454, T476, S581, S597) have been mapped on human Gab1 in assays in which recombinant Gab1 was subject to an *in vitro *phosphorylation reaction using recombinant ERK1/2 [173]. All these sites are located within putative MAPK phosphorylation motifs (Fig. 5). Interestingly, most studies pinpoint towards a negative role for ERK in Gab1 signalling as an increase in the Ser/Thr-phosphorylation content of Gab1 is correlated with a decrease in its tyrosine phosphorylation [90,168,171,174]. Although the molecular mechanisms involved in ERK-mediated inhibition of Gab1 tyrosine phosphorylation still remain ill-defined, it should be mentioned that four of these sites (S454, S581, S597, T476) are located within the vicinity of the YVPM motifs involved in p85 recruitment [171,173,174]. However, a positive role for the ERK-mediated feedback phosphorylation of Gab1 has been also described [172]. Furthermore, Eulenfeld and Schaper (2008) have revealed that an additional MAPK-dependent phosphorylation site in Gab1, S552, modulates the function of the PH domain in a positive manner and thereby contributes to the IL-6-mediated recruitment of Gab1 to the plasma membrane [175]. Although the precise molecular mechanism remains to be elucidated, this study suggests that phosphorylation may regulate Gab proteins *via *conformational change. Lastly, Gab1 has been shown to be a substrate of the Ser/Thr-kinase ROK *in vitro *and possibly *in vivo*, although the sites of phosphorylation and the functional consequences of these phosphorylation events remain to be identified [176].

### Gab2 is the target of several negative feedback loops

Gab2 is also subject to Ser/Thr-phosphorylation at multiple sites (Fig. 5). In this regard, we have recently identified 21 novel phosphorylation sites on Gab2 purified from growth factor-stimulated mammary epithelial cells [49]. The recent tally in Phosphosite http://www.phosphosite.org currently lists 10 tyrosine, 18 serine and 5 threonine *bona fide *phosphorylation sites indicating that Gab2 is a heavily phosphorylated protein.

In 2002, Lynch and Daly reported that Gab2 is phosphorylated within a typical AKT phosphorylation motif encompassing S159 [177]. Furthermore, this study showed that the prominent growth factor-induced electrophoretic mobility shift of Gab2 is mediated by both PI3K- and MEK-dependent feedback loops. Importantly, inhibition of the PI3K/AKT pathway or mutation of S159 resulted in increased tyrosine phosphorylation of this docking protein, and the Gab2^S159A ^mutant displayed transforming properties in fibroblasts. To our knowledge, this was the first evidence that the oncogenic potential of docking proteins can be harnessed by negative feedback control. This concept was subsequently supported by a study from the Schlessinger laboratory showing that the negative feedback phosphorylation of FRS2α by ERK suppresses its transforming potential [13]. In agreement with the original findings by Lynch and Daly (2002), a recent study comparing murine breast cancers driven by an ErbB2 transgene alone or in combination with an constitutively activated AKT transgene showed that the phosphorylation of Gab2 at Y452 was dramatically reduced in the latter [178]. Although, the phosphorylation status of S159 was not addressed in this study, it is tempting to speculate that the aforementioned Akt-mediated feedback loop is responsible for the attenuation of Gab2 tyrosine phosphorylation. Another recent report has identified the Ser/Thr-phosphatase calcineurin as novel interaction partner of Gab2 that interacts with the serine-rich region C-terminal of the PH domain [179]. This region includes S159 and ectopic expression of calcineurin results in reduced recognition of Gab2 by an anti-AKT substrate antibody, which appears to detect predominantly pS159. In line with the negative role of S159 [177], co-expression of Gab2 and a catalytically active, but not a phosphatase-dead, form of calcineurin enhanced IL-3 mediated activation of a *c*-*fos*-reporter construct in a synergistic manner. As described later in this section, Gab2 is also subject to additional PI3K-dependent negative feedback events on S210 and T391 [49].

Gab2 is also regulated by ERK-mediated negative feedback phosphorylation, as identified by Arnaud *et al*. in 2004 in IL-2 stimulated T lymphocytes [80]. Previously, ERK or an ERK-dependent kinase had been implicated in the phosphorylation of Gab2 [177,180], however, the phosphorylation site(s) was not characterised. Arnaud *et al *(2004) identified S623 as the site of action of this ERK-mediated feedback loop and provided evidence that the Gab2/SHP2 interaction is enhanced by a S623A mutation.

Taken together, a series of studies over the last decade has shown that the tyrosine phosphorylation and signalling potential of docking proteins such as those of Gab, IRS, FRS and SLP families is counteracted by their Ser/Thr-phosphorylation, which usually represents the endpoint of feedback loops from cytoplasmic signalling cascades [13,15,49,81,169,177,181]. Consequently, major tasks for the future will be to characterize the spatiotemporal regulation of these phosphorylation events in response to specific stimuli, the kinases and phosphatases involved and the mechanisms by which such modifications control signal output.

### How is Gab function regulated by feedback phosphorylation?

**Figure 6:**
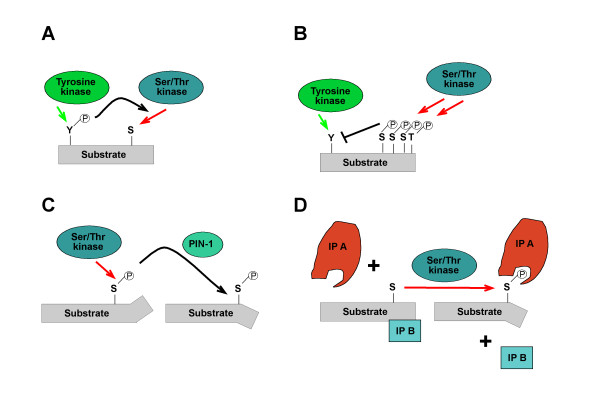
**Potential functional effects of Gab phosphorylation**. **(A/B) **Phosphorylation of one residue affects the phosphorylation of a nearby residue in either a positive or antagonistic fashion. **(C) **Phosphorylation-induced conformational changes by the *cis*/*trans *peptidyl-prolyl-isomerase PIN-1. **(D) **Phosphorylation affects the composition of multi-protein complexes, e.g. by phosphorylation-induced conformational changes or the creation of docking sites, e.g. for proteins with SH2, PTB, or WW domains or for 14-3-3 proteins. IP A and IP B stand for interaction partners A and B, respectively. For details refer to text.

Potential mechanisms that may underpin the action of phosphorylation-dependent positive or negative feedback on Gab proteins are summarized in Fig. 6. Firstly, phosphorylation of a particular residue might affect the phosphorylation of a nearby residue in either a positive or antagonistic fashion, due to phosphorylation-induced changes in protein conformation or simply changes in the electrostatic landscape of the substrate protein [182] (Fig. 6A/B). Secondly, phosphorylation-induced conformational changes may alter the accessibility of key regions, such as the PH domain. These may occur due to electrostatic repulsion/attraction between distinct protein moieties or phosphorylation-induced *cis*/*trans *peptidyl-prolyl-isomerisation (Fig. 6C). Although Gab proteins have not been identified as substrates of peptidyl-prolyl-isomerases such as PIN1 yet [183], the high number of phosphorylation sites preceding proline residues and the fact that Gab proteins are targeted by Pro-directed kinases such as ERK support the likelihood of this regulatory mechanism [80,173,175].

A third mechanism by which docking proteins can be negatively regulated by protein phosphorylation is *via *changes in their "social behaviour", specifically alterations in their ability to interact with crucial interaction partners or in their subcellular localisation (Fig. 6D; [4]). Key mediators of this kind of mechanism are 14-3-3 proteins, a highly- conserved and ancient group of eukaryotic adaptor proteins that bind to specific phospho-Ser/Thr-residues in their client proteins and thereby execute the effect of phosphorylation events, either by stabilizing certain protein conformations or regulating intermolecular protein-protein interactions [2]. Several docking proteins such as KSR, SLP-76 and IRS proteins have been described as 14-3-3 client proteins [4,15,181] and we recently reported that Gab2 interacts with 14-3-3 proteins in a phosphorylation-dependent manner [49]. This interaction is mediated by two 14-3-3 binding motifs surrounding S210 and T391 that flank the typical Grb2 binding site. Interestingly, while Akt phosphorylates Gab2 only at S159 [177], the phosphorylation of S210 and T391 is attenuated by PI3K and AKT inhibitors indicating that the responsible Ser/Thr-kinases are positively modulated by the PI3K-AKT axis and are therefore acting in negative feedback mode [49]. In support of this model, Gab2 mutants defective in 14-3-3 binding exhibit increased recruitment of Grb2 and consequently sustained association with the tyrosine phosphorylated EGFR and Shc. Furthermore, these Gab2 mutants promote cellular proliferation and transformation. Conversely, introduction of constitutive 14-3-3-binding sites into Gab2 drastically reduces its ability to recruit Grb2 and renders it refractory to receptor activation, demonstrating that site-selective binding of 14-3-3 proteins is sufficient to terminate Gab2 signalling. Based on these findings, we proposed a model in which signal attenuation occurs, because 14-3-3 promotes dissociation of Gab2 from Grb2, and thereby uncouples Gab2 from the receptor complex. As shown in Figs 2 and 3, the Gab2/Grb2 interaction is pivotal for the recruitment of this docking protein to most, if not all receptors and consequently this novel regulatory mechanism should have broad implications for diverse signalling systems. Interestingly, the 14-3-3 recruitment motifs around S210 and T391 are conserved in Gab2 orthologues from bony fish to mammals, but are absent from Gab1 and Gab3 paralogues. Gab4 contains the 14-3-3 binding motif around S210, but lacks the motif around T391 and the typical Grb2 binding site, which is positioned in N-terminal vicinity of T391. Furthermore, these motifs are also absent from DOS and SOC-1 suggesting that the 14-3-3 interaction is a vertebrate-specific regulatory layer for Gab2. However, Scansite [121] predicts three potential mode I 14-3-3 binding sites in NeGab (S162, S328 and T516), which also flank the equivalent of the typical Grb2 binding site in NeGab (359-366). Although this remains purely speculative, this observation could indicate that the Gab/14-3-3 interaction is an ancestral feature that was modified or lost during the evolution of the SOC-1, DOS and Gab1/3 proteins.

## Gab docking proteins in human pathologies

### Gab proteins and tumourigenesis

Given their pivotal role in many physiological processes, it is perhaps not surprising that Gab proteins are implicated in a variety of human diseases. In particular, Gab proteins contribute to aberrant PTK signalling in certain malignancies, reflecting their functions as signal amplifiers. Only a few mutations have been reported in human Gab proteins so far [184] and COSMIC database) and due to their low frequency it is unclear whether the corresponding mutant Gabs represent real drivers or merely passengers of tumourigenesis. However, it is well-established that Gab proteins promote tumourigenesis by functioning as 'accomplices' of certain oncoproteins or by amplifying signalling upon their overexpression. In the following sections we will provide an update regarding their identified roles in hematopoietic disorders and solid tumours.

#### Haematological neoplasia

The first evidence for the critical involvement of Gab2 in leukemogenesis was the groundbreaking finding that myeloid progenitors from Gab2-deficient mice are resistant to transformation by Bcr-Abl [185]. The latter represents a leukemogenic fusion protein generated as a consequence of a chromosomal translocation found in more than 90% of patients with chronic myeloid leukaemia (CML). Phosphorylation of Y177 within the Bcr moiety leads to recruitment of the Grb2/Gab2 complex and downstream signalling *via *SHP2 and PI3K, which is crucial for enhanced proliferation and survival. Similarly, the oncogenic Bcr-FGFR1 fusion protein, which is also the product of a chromosomal translocation (Table 1) and consists of a Bcr-derived moiety and the tyrosine kinase domain of the fibroblast growth factor receptor 1 (FGFR1), drives the tyrosine phosphorylation of Gab2 in murine bone marrow cells and their malignant transformation through phospho-Y177 mediated Grb2 association [186]. These data strongly suggest that Grb2-mediated recruitment of Gab2 to oncogenic fusion protein tyrosine kinases is a critical event for the induction of a CML-like disease.

**Table 1:** Oncogenic events in human and murine leukemias involving Gab2

Genetic aberration	Leukemic disease	Involvement of Gab2	References
Bcr-Abl translocation t(9;22)	CML, B-ALL	Recruitment of Grb2/Gab2 complex to Y177 of Bcr-Abl Y177 and Gab2 are essential for Bcr Abl-mediated transformation and leukemogenesis	[185,187]
Bcr-FGFR1 t(8;22)	CML-like disease	Recruitment of Grb2 and presumably Gab2 to Y177 of Bcr-Abl. Increased Gab2 tyrosine phosphorylation	[186]
Tel-Abl translocation t(9;12)	B-ALL, T-ALL, CML	Recrutiment of Grb2/Gab2 complex to Y314 of Tel-Abl Y314 is essential for Tel Abl-mediated transformation and leukemogenesis	[191]
Tel-Jak2 translocation t(9,12)	ALL	Some isoforms of Tel-JAK2 recruit the Grb2/Gab2 complex via Y314	[190]
Npm-Alk translocation t(2;5)	Anaplastic large cell lymphomas	Gab2, SHP2 and Grb2 form a complex with Npm-Alk	[242]
SHP2 E76K point mutation	JMML	E76K mutation confers enhanced catalytic activity to SHP2 and requires Gab2 for transformation	[200]
Sf-Stk	Friend's virus-induced erythroleukemia in mice	Recruitment of the Grb2/Gab2 complex to Sf-Stk is essential for erythroid transformation by Friend virus, this involves the direct binding of STAT3 to Gab2	[36,81]
Amplification of MLL locus	AML/MDS	Gab2 is frequently co-amplified with the mixed lineage leukaemia (MLL) gene	[210]

The pivotal role of Gab2 in Bcr-Abl signalling is further underscored by the observation that shRNA-mediated silencing of endogenous Gab2 inhibits proliferation and colony formation of CD34^+ ^cells from CML patients, but not their counterparts isolated from healthy donors [187]. However, the role of Gab2 in CML might be more complex than just driving proliferation and survival through the PI3K and SHP2/Ras pathways. Indeed, through the recruitment of SHP2, Gab2 tightly controls ERK/MAPK signalling, which will, if it exceeds a certain threshold, drive the terminal differentiation rather than the proliferation of Bcr-Abl transformed myeloid progenitors. Indeed, Gab2 over-expression induces increased ERK activation and megakaryocytic differentiation of the CML cell line K562 [188]. This suggests that the expression levels and signalling competence of Gab2 needs to be tightly controlled in Bcr-Abl^+ ^CML in order to drive proliferation and to repress differentiation at the same time, raising the possibility that modulation of Gab2 signalling might represent a strategy to control this disease.

Despite the great clinical success of the PTK inhibitor imatinib in the therapy of CML, imatinib resistance, due to acquired mutations in the Bcr-Abl oncogene or subsequent alterations in the cellular signalling network, remains a serious clinical challenge [189]. Interestingly, imatinib resistance in the absence of detectable Bcr-Abl kinase mutation is often mediated by persistent activation of the Src family kinase Lyn, which tyrosine phosphorylates Gab2 leading to activation of its downstream effectors. Lyn inhibition silences Gab2 and Bcr-Abl tyrosine phosphorylation and restores imatinib sensitivity [86]. Another kinase implicated as a key component of the Bcr-Abl signalling network is Jak2 that in turn activates Lyn leading to Gab2 phosphorylation. Consequently, pharmacological or siRNA-mediated inhibition of Jak2 or Lyn reduces tyrosine phosphorylation of Gab2 in CML cells. Taken together, these findings identify Jak2 and Lyn as additional drug targets in CML and further highlight the important role of tyrosine-phosphorylated Gab2 as a driver of CML [86,91,95].

After the pivotal role of Gab2 in Bcr-Abl-mediated transformation had been established, its involvement in the pathogenesis of several other leukemias was discovered (Table 1). The oncogenic fusion kinases Tel-Abl and Tel-Jak2 engage Gab2 in a similar manner to Bcr-Abl [190,191]. Tyrosine 314 is crucial for the recruitment of the Grb2/Gab2 complex to Tel-Abl and presumably to Tel-Jak2 as well [192]. Consequently, a Tel-Abl^Y314F ^mutant exhibits reduced fibroblast transforming capacity and fails to induce a CML-like disease in mice [191]. It should be emphasised that the common denominator of the structurally unrelated Bcr and Tel fusion partners is their potential to recruit Grb2/Gab2 complexes, which underscores again the significance of Gab2 as an amplifier of dysregulated signalling by Abl, Jak2 and FGFR1.

#### The role of Gab proteins in JMML and NCFC syndromes

Juvenile myelomonocytic leukemia (JMML) and the neuro-cardio-facious cutaneous syndromes (NCFC) are human pathologies caused by aberrant Ras/ERK signalling. The NCFC syndromes comprise neurofibromatosis (NF) and the Noonan (NS), Costello (CS), LEOPARD (LS) and cardio-facious-cutaneous (CFC) syndromes, which are correlated with autosomal-dominant germ-line mutations within either the core components (Ras, B-Raf, Raf-1, MEK) or modulators of the Ras/ERK pathway (NF1, SHP2, SOS and Spred). The resulting mutant proteins display aberrant activities and consequently disturb the overall fine-tuning of the Ras/ERK pathway and to a certain degree the Ras/PI3K pathway [35,193]. As the ERK pathway steers both proliferation and differentiation, many processes underlying normal human development and organ homeostasis are perturbed and give rise to the various clinical symptoms, which range from cardiac defects, skin and cranio-facial abnormalities to growth and mental retardation [194-196]. Importantly, some NCFC syndromes predispose affected individuals to neoplastic diseases [196]. Indeed, the discovery of germline missense mutations in the SHP2-encoding *PTPN11 *gene in ~50% of NS cases led to the identification of *PTPN11 *as the most common target of somatic mutations in JMML, a rare, albeit aggressive myelo-proliferative disorder occurring in children, where *PTPN*11 mutation rates of up to 35% have been reported [196-199]. The most frequently JMML-associated mutation, E76K, confers enhanced catalytic activity to SHP2 and requires Gab2 for transformation of primary murine myeloid progenitors [200]. However it should be noted that the nature of somatic JMML-associated *PTPN11 *mutations differ from the germline mutations identified in Noonan syndrome in that JMML-associated *PTPN11 *alleles usually encode stronger gain-of-function mutant proteins [200,201]. Nevertheless, this finding demonstrates that Gab2 is an important player in JMML and suggests that NS-associated SHP2 mutants may require Gab proteins as recruitment devices in a similar manner. Indeed, co-expression experiments in COS-7 cells revealed that NS-associated SHP2 mutants exhibit a stronger and more sustained interaction with Gab1 than SHP2^wt^. Importantly, co-expression of Gab1^ΔSHP2 ^in this system blocks the EGF-induced increase in the phosphatase activity of the NS-associated SHP2 mutants and consequently abolishes their positive effect on EGF-induced ERK phosphorylation [202].

While NS patients carry mostly gain-of-function mutations in SHP2, this phosphatase often contains dominant-negative mutations in LS patients [197,203]. Interestingly, expression of LS-associated SHP2 mutants with impaired catalytic activity in cells strongly enhances the EGF-induced interaction between Gab1 and p85 [161], which suggests that these mutant proteins, while acting in dominant negative fashion on the Ras/ERK pathway, may promote aberrant PI3K activation by protecting the p85 recruitment sites against SHP2^wt^. Taken together, these studies identify Gab proteins as important "accomplices" of NCFC-associated SHP2 mutants and suggest that a better knowledge of Gab signalling will contribute to an improved understanding and treatment of these syndromes. Furthermore, the close relationship between SHP2 and Gab proteins and the important role of Gab proteins as modulators of Ras signalling also raise the question as to whether the Gab genes themselves are awaiting their identification as novel "NCFC" alleles.

#### Aberrant activation and/or expression of Gab proteins in solid tumours

Dysregulated Gab signalling is also increasingly recognised as an important contributor to the biology of solid tumours. Firstly, the signalling potential of Gab1 needs to be considered in tumours with aberrant expression or mutations of c-MET [17,204]. As discussed above several studies have demonstrated the close collaboration between c-Met and Gab1 and, in contrast to many other RTKs that merely induce a transient tyrosine-phosphorylation of Gab1, c-MET induces a very sustained tyrosine phosphorylation of this docking protein [24,129]. Furthermore, a recent study has revealed Gab1 not only as a convergence point between c-MET and EGFR pathways, but also suggests that Gab1 cooperates with *MET *amplification in lung cancer cells, which have become resistant towards the EGFR inhibitor gefitinib [204]. A correlation between the tyrosine phosphorylation status of Gab1 and the progression of ErbB2-transgene driven murine mammary tumours has also been reported, indicating that Gab1 needs to be considered as an important downstream effector of this oncogenic RTK as well [205]. Lastly, it should be mentioned that somatic missense mutations resulting in conversion of the amino acid residues Y83, T387 and R498 into C, N and M, respectively, have been identified in the human *GAB1 *gene in human breast and lung cancers, albeit at very low frequencies (for details see the COSMIC database at http://www.sanger.ac.uk/genetics/CGP/cosmic/ and Ref. [184]). However, it remains to be tested as to whether these mutations alter the signalling properties of Gab1, and so it is unclear at this stage whether they represent real drivers or merely passengers of tumourigenesis.

In contrast to Gab1, Gab2 is developing a strong track record as an oncoprotein in its own right in various solid tumours. Firstly, Gab2 is frequently over-expressed in human breast cancer cell lines and primary tumours and becomes tyrosine phosphorylated in these cells in response to EGF, insulin and bFGF stimulation [163]. This indicates that a variety of RTKs implicated in breast cancer development or progression use Gab2 to amplify their signals. It should be mentioned that there might be several, not necessarily mutually exclusive mechanisms by which Gab2 is up-regulated in breast cancer such as the amplification of the *GAB2 *locus on 11q13-14 [35], a region commonly amplified in breast cancers, the aberrant activity of the E2F transcription factor [162,206], which is often dys-regulated in tumours and binds directly to the human *GAB2 *promoter, and aberrant estrogen receptor (ER) signalling [163]. Indeed, the original study on Gab2 in breast cancer demonstrated that the expression of both Gab2 mRNA and protein was induced by estradiol in an ER-dependent manner [163]. These observations spurred investigations in several laboratories as to whether the over-expression of Gab2 represents a cause or consequence of tumour development. In order to address this question, the Daly and Neel laboratories made use of the immortalised, but non-transformed human mammary epithelial cell MCF-10A, which expresses very low levels of Gab2 [163] and generates acinar structures in three dimensional (3D) matrigel cultures. Consequently, this cell line is frequently used to characterise the impact of oncogenes on hallmarks of epithelial development and transformation [207]. In the first study, Brummer *et al*. applied a bi-cistronic retroviral expression system to adjust the Gab2 expression in MCF-10A cells to levels observed in human breast cancer cell lines and analysed the intracellular signalling events in these cells [39]. In monolayer culture, overexpression of Gab2 accelerated EGF-induced cell cycle progression and was associated with enhanced and/or more sustained EGF-induced ERK and AKT activation. When grown in 3D matrigel culture, MCF-10A cells expressing ectopic Gab2 were still able to generate polarized, growth-arrested acini with hollow lumina. However, the acini were larger due to increased cell proliferation, and the suppression of proliferation that normally occurs in late 3D stage cultures was attenuated [39]. Very similar findings were independently reported by the Neel laboratory [35]. The effect of Gab2 on acinar size was dependent on the presence of intact Grb2 and SHP2 binding motifs and was enhanced by its potential to recruit PI3K [39]. Importantly, Gab2 also conferred independence of the morphogenetic program from exogenous EGF and intrinsic EGFR kinase activity [39].

Amplification and/or over-expression of the human *GAB2 *gene has been also recently reported for ovarian [208] and gastric cancer [209] and acute myeloid leukemia (AML) [210], although additional functional studies are required to dissect the role that Gab2 plays in these malignancies. Furthermore, two recent studies in melanoma support the findings from the aforementioned breast cancer models in various aspects [211,212]. Firstly, Horst *et al*. have shown that, similar to breast cancers and other neoplasias, the *GAB*2 gene is amplified and/or over-expressed in 11% and 50% of human metastatic melanomas, respectively [211]. Moreover, Chernoff *et al*. (2009) demonstrated that *GAB*2 amplification is associated with melanoma arising from sun-protected sites and often occurs independently from oncogenic *NRAS *or *BRAF *mutations or amplification of the *KIT *gene [212]. Importantly, knockdown and over-expression experiments revealed that Gab2 enhances the migratory and invasive behaviour of melanoma cells in a PI3K-dependent manner [211]. In contrast to the over-expression of Gab2 in metastatic melanoma, normal human melanocyte lines, melanocytic nevi and primary melanomas displayed low Gab2 expression levels suggesting that Gab2 overexpression might represent a marker of neoplastic progression [211].

#### Cooperation of Gab2 with other oncogenes in solid tumours

We have previously reported that MCF-10A cells expressing very high levels of Gab2 generate large disorganized structures in 3D culture with defective luminal clearance [39], a phenotype that is frequently observed in this system upon ectopic expression of activated RTKs [207,213]. Although it is uncertain at this stage as to whether such high Gab2 expression levels occur in breast cancers, these data underscore the oncogenic potential of Gab2 and suggest that Gab2, although being a weak oncogene by itself, might be an important cooperation partner of other oncoproteins. Indeed, such a cooperation of Gab2 with other oncoproteins has been previously demonstrated with Sf-STK, v-Sea and polyoma middle T antigen [36,214,215]. Furthermore, the Neel laboratory could demonstrate that coexpression of Gab2^wt^, but not Gab2^ΔSHP2^, with the RTK Neu (also known as ErbB2 and HER2) resulted in an invasive growth phenotype of MCF-10A cells in 3D culture [35]. Importantly, this study also showed that NeuNT-transgene-evoked mammary tumourigenesis is potentiated or reduced in MMTV-Gab2 transgenic and Gab2-deficient mice, respectively.

The studies of Bentires-Alj *et al*. [35] were complemented by a recent report from the Feng laboratory demonstrating that ablation of Gab2 severely suppresses lung metastasis of Neu-induced mammary tumours and that Neu-transformed but Gab2-deficient mammary epithelial cells exhibit decreased migration and impaired ERK activation, [216]. Here, the authors could show that Gab2 expression levels were elevated in mammary tumours induced by the Neu (ErbB-2) oncogene suggesting that, as discussed above, an oncoprotein-distorted signalling network alone might be sufficient to up-regulate the expression of Gab2, e.g. *via *increased E2F activity. However, Ke *et al*. reported that loss of Gab2 in mice had only a modest effect on the initiation and growth rate of mammary tumours induced by a constitutively active *neu *transgene (Neu2-5) or a signalling-compromised version, NeuYD, which can only recruit Shc proteins [216]. There are two potential explanations for differences in the results of this study to those from the Neel laboratory [35]. Firstly, the studies used independent Gab2-deficient mouse strains generated by different *knock-out *strategies, with one strain expressing low amounts of a N-terminally truncated Gab2 protein [112]. Secondly, intrinsic differences between the NeuNT, Neu2-5 and NeuYD transgenes used might account for the observed differences in tumour onset and growth. Despite these discrepancies, it is clear that Gab2 co-operates with Neu to promote the development or progression of mouse mammary tumours. Interestingly, the requirement for a Gab docking protein for the efficient action of an activated Neu/ErbB2 is not restricted to mammalian systems as DOS cooperates with a *neu *transgene in *Drosophila *[217].

The cooperation of Gab2 with oncoproteins in solid tumours is not restricted to oncogenic RTKs such as ErbB2 and v-Sea. For example, the non-receptor tyrosine kinase c-Src is often aberrantly expressed or activated in human breast cancers, sometimes as a consequence of dys-regulated ErbB2 activity [218,219]. As the tyrosine phosphorylation status of Gab2 is regulated by members of the Src family, Bennett *et al*. (2008) investigated the biological consequences of the co-expression of Gab2 and Src proteins in the aforementioned MCF-10A model [94]. This study demonstrated that, while over-expression of c-Src by itself did not affect acinar morphogenesis or growth factor dependence in 3D culture, c-Src co-operated with Gab2 to promote EGF-independent acinar growth. Furthermore, Gab2, but not Gab2^Δp85^, significantly enhanced acinar disruption induced by the hyper-active v-Src and c-Src^Y527F ^mutants [94]. This phenotype was associated with a significant reduction in the adhesive strength of E-Cadherin, a cell adhesion molecule critical for acinar morphogenesis, without altering its surface expression. Furthermore, Gab2 associated with E-Cadherin in the presence and absence of v-Src, indicating that the ability of Gab2 to weaken the strength of cell-cell contacts may reflect enhanced activation of PI3K at adherens junctions. It should be noted that Gab2 also increased migration and invasion of MCF-10A cells expressing activated Src proteins, but these effects were p85-independent and might be mediated by the SHP2 effector branch.

Lastly, as Gab2 is an important amplifier of PI3K signalling, it is tempting to speculate that Gab2 overexpression might cooperate with the *BRAF*^V600E ^oncogene in melanoma. The V600E mutation is a very frequent and early-arising event in the nevi-melanoma progression series but, by itself, induces only a transient enhancement of proliferation followed by cell cycle arrest with hallmarks of cellular senescence [220]. Indeed, a recent study involving conditional mouse models has shown that *BRAF*^V600E ^cooperates with the loss of PTEN in the induction of metastatic melanomas [221], which underscores the idea that *BRAF*^V600E ^requires increased levels of PI3K activity to drive malignant melanomas. Thus, Gab2 might even cooperate with oncogenes that are not directly associated with the Gab2 signalosome.

In summary, a series of studies conducted in various experimental settings have now demonstrated that Gab2 is not only an important interaction partner of oncoproteins involved in the transformation of hematopoietic cells, but also of those playing a well-described role in solid tumours. It appears likely that more co-operating oncogenes for Gab2 in solid tumours will be found in the not too distant future. One of these candidates might be again SHP2, which is mutated at low frequency in AML and several solid cancer types and commonly overexpressed in breast cancer, where it is involved in regulating epithelial/mesenchymal transition [222-224]. Studies to date demonstrate that Gab2 can promote the proliferation, growth factor autonomy, migration and invasion of cancer cells, indicating that it may contribute to several stages of tumour progression. An important avenue for further research will be to identify whether Gab2 associates with patient prognosis or therapeutic responsiveness in particular malignancies, such as breast cancer.

### Molecular mimicry of Gab proteins

The CagA protein of the gastric pathogen *Helicobacter pylori *is translocated into gastric epithelium cells of the host where it interacts with Grb2, becomes tyrosine phosphorylated and recruits effectors such as SHP2 and Crk to enhance Ras/ERK signalling and cellular transformation [225-228]. However, despite these functional similarities to Gab proteins, CagA shares no sequence homology with members of the Gab/DOS family, indicating that it functions *via *molecular mimicry of these eukaryotic docking proteins. Given the association between CagA and development of gastric carcinoma [225], an interesting possibility is that the aforementioned overexpression of Gab2 in gastric cancer [209] triggers similar events in the gastric epithelium to *Helicobacter *infection and that the gastric epithelium is susceptible towards transformation by aberrant SHP2 activity. Interestingly, this concept of molecular mimicry is now strongly supported by recent experiments in *Drosophila *demonstrating that a *cag*A transgene can rescue larval viability and photoreceptor development in mutant animals lacking DOS [229]. Furthermore, an epistasis analysis also revealed that the DOS complementing function of CagA requires the expression of the SHP2 orthologue CSW.

### Is Gab2 involved in Alzheimer's disease?

In addition to the various neoplastic diseases, Gab2 is also increasingly implicated in Alzheimer's disease (AD). Reiman *et al*. (2007) identified certain *GAB2 *alleles as modifiers of disease susceptibility in carriers of the APOEε4 allele that is strongly associated with late-onset AD [230]. Although other independent studies failed to replicate this association [231-233], the initial findings were confirmed by Belgian and Italian studies [234,235] and recent meta-analyses suggest that there is indeed a significant association between GAB2 allelic variation and AD risk [236,237]. It remains to be elucidated how these SNPs affect Gab2 expression and/or function. However, Reiman *et al*. (2007) could demonstrate that siRNA-mediated reduction of Gab2 expression in neuroglioma cells results in increased Tau protein phosphorylation at Ser 262, a residue, which is hyper-phosphorylated in AD and has been implicated in neurofibrillary tangle formation. As this residue is targeted by GSK-3 and this kinase is inhibited by AKT-mediated phosphorylation [238], this finding is consistent with the well-established function of Gab2 as an amplifier of PI3K/AKT signalling. Clearly, more genetic data and in particular functional analyses will be required to deliver a verdict on the role of Gab2 in AD.

## Conclusion and perspectives

Since their discovery, Gab docking proteins have emerged as critical players in many physiological processes as well as pathologies such as cancer and inflammatory diseases. It is becoming more and more evident that their versatile roles in signal transduction extend beyond the original and relatively static definition of a docking protein. We are starting to appreciate that docking proteins play a central role in the management of entire signalling subsystems and that they are, at the same time, subject to complex spatiotemporal control by the same network, e.g. *via *phosphorylation events. They orchestrate multiple protein-protein and -lipid interactions and also act as allosteric activators. The diversity of Gab interaction partners also implies that there are distinct types of Gab signalosomes present in the cell, which differ in their subcellular localisation and function. Thus, more refined biochemical approaches will be required to characterize the composition and stoichiometry of the different Gab signalosomes. It is also becoming evident that Gab proteins mediate the crosstalk between various signalling pathways and thereby provide the basis for the synergistic action of various receptors [154,204,239], which reflects the real *in vivo *situation as the cells in our bodies are simultaneously exposed to a plethora of biologically-active ligands. However, in order to fully understand the signalling roles of Gab proteins, it is clear that various disciplines will need to cooperate and utilize a systems-based approach that integrates structural and biophysical studies on regulation of protein-protein interactions, mathematical and computational modelling of the Gab signalling network and functional analyses that exploit the genetics of appropriate model organisms. Such an endeavour is likely to provide exciting new insights into the mechanisms and functions of Gab signalosomes.

## List of abbreviations

Aa: Amino acid; AD: Alzheimer's disease; AML: Acute myeloid leukemia; BCR: B cell antigen receptor; Bcr: Breakpoint cluster region; bFGF: Basic fibroblast growth factor; BMMCs: Bone marrow derived mast cells; BTAM: Bi-phosphoryl tyrosine activation motif; BTK: Bruton's tyrosine kinase; CBP: Csk-binding protein; CD: Cluster of differentiation; CFC: Cardio-facious-cutaneous syndromes; CML: Chronic myeloid leukemia; CNS: Central nervous system; Crk: Sarcoma virus CT10 oncogene homolog; CS: Costello syndrome; CSF: Colony stimulating factor; CSW: Corkscrew; DAG: Diacylglycerol; DNA: Deoxyribonucleic acid; DOS: Daughter of sevenless; EGF: Epidermal growth factor; ERK: Extracellular signal regulated kinase; EST: Expressed sequence tag; EPO: Erythropoietin; FGF: Fibroblast growth factor; FRS: Fibroblast growth factor receptor substrate; Gab: Grb2-associated binder; GADS: Grb2-related adaptor downstream of Shc; GAP: GTPase activating protein; Grb2: Growth factor receptor-bound protein 2; Gsk: Glycogen synthase kinase; GST: Glutathione S-transferase; GTP: Guanine nucleotide trisphosphate; HER: Human epidermal growth factor receptor; HGF: Hepatocyte growth factor; IL: Interleukin; IP_3_: Inositoltrisphosphate; IRS: Insulin receptor substrate; JAK: Janus kinase/Just another kinase; JMML: Juvenile myelomonocytic leukemia; kDa: Kilodalton; KSR: Kinase suppressor of ras; LAT: Linker of activated T cells; LPA: Lysophosphatidic acid; LS: LEOPARD syndrome (multiple lentigines: electrocardiographic conduction defects; ocular hypertelorism; pulmonary stenosis; abnormalities of the genitalia; retardation of growth and sensorineural deafness); MAPK: Mitogen activated protein kinase; MBD: Met binding domain; MEFs: Mouse embryonic fibroblasts; MEK: Mitogen activated protein/extracellular signal regulated kinase kinase; MONA: Monocytic adaptor; NCFC: Neuro-cardio-facious-cutaneous syndromes; NF: Neurofibromatosis; NF-κB: Nuclear Factor kappa B; NGF: Nerve growth factor; NK: Natural killer; NRG: Neuregulin; NS: Noonan syndrome; PAG: Phosphoprotein associated with glycosphingolipid-enriched microdomains; PAK: p21-activated kinase; PDGF: Platelet-derived growth factor; PH: Pleckstrin homology; PI3K: Phosphatidyl-inositol-3 kinase; *PI3KCA*: gene encoding the catalytic subunit of phosphatidyl-inositol-3 kinase; PIP: Phosphatidyl-inositol-phosphate; PKB: Protein kinase B; PLC: Phospholipase; PTB: Phospho-tyrosine binding; PTEN: Phosphatase and Tensin homolog; PTK: Protein tyrosine kinase; PTP: Protein tyrosine phosphatase; PTPN: Protein tyrosine phosphatase: non-receptor; Raf: Rapidly growing fibrosarcoma; RANK: Receptor Activator of NF-κB; Ras: Rat sarcoma; RNA: Ribonucleic acid; RNAi: RNA interference; ROK: Rho kinase; RTK: Receptor tyrosine kinase; SCF: Stem cell factor; Sea: S13 erythroblastosis oncogene homolog; siRNA: Small interfering RNA; SH2: Src homology 2; SH3: Src homology 3; SHIP: SH2-containing inositol 5-phosphatase; SHP: SH2 domain-containing protein-tyrosine phosphatase; SLP: SH2 domain containing leukocyte protein; SOC: Suppressor of clear; Src: Sarcoma viral oncogene homolog; STAT: Signal transducer and activator of transcription; Syk: Spleen tyrosine kinase; ZAP-70: Zeta-chain associated protein of 70 kDa.

## Competing interests

The authors declare that they have no competing interests.

## Authors' contributions

FUW, RJD and TB surveyed and selected the literature for this review. FUW prepared the figures and tables with input from her co-authors. All three authors were involved in writing the manuscript, which was compiled by TB All three authors read and approved the final manuscript.
